# Continuing education to combat domestic and gender-based violence: an experience report

**DOI:** 10.1590/0034-7167-2024-0338

**Published:** 2025-08-08

**Authors:** Maria Fernanda Terra, Rosa Maria Godoy Serpa da Fonseca

**Affiliations:** I Universidade de São Paulo. São Paulo, São Paulo, Brazil

**Keywords:** Nursing Supervision, Nursing, Gender Equity, Violence Against Women, Education, Continuing, Supervisión de Enfermería, Enfermería, Equidad de Género, Violencia contra la Mujer, Educación Continua

## Abstract

**Objectives:**

to report the implementation of supervision for healthcare professionals from Violence Prevention Centers in healthcare services in two regions of the city of São Paulo, Brazil.

**Methods:**

an experience report of planning and executing supervision meetings from the perspective of critical-emancipatory education.

**Results:**

twenty meetings were held with critical reflections on domestic and gender-based violence as a barrier to continuity of life and the need to make the phenomenon visible and take it into account when planning healthcare.

**Final Considerations:**

the experience offers a practical perspective on supervising health workers that is consistent with maintaining ongoing education and health surveillance to improve the way domestic and gender-based violence is addressed in healthcare services.

## RESUMEN

**Objetivos:**

informar la implementación de la supervisión de los profesionales de la salud en los Centros de Prevención de la Violencia en los servicios de salud de dos regiones de la ciudad de São Paulo, Brasil.

**Métodos:**

relato de experiencia sobre la planificación y ejecución de reuniones de supervisión desde la perspectiva de la educación crítico-emancipadora.

**Resultados:**

se realizaron 20 encuentros con reflexiones críticas sobre la violencia doméstica y de género como barrera para la continuidad de la vida y la necesidad de visibilización y consideración del fenómeno en la planificación de la atención en salud.

**Consideraciones Finales:**

la experiencia ofrece una perspectiva práctica sobre la supervisión de los trabajadores de la salud consistente con el mantenimiento de una educación y vigilancia sanitaria continua para mejorar la lucha contra la violencia doméstica y de género en los servicios de salud.

## INTRODUCTION

The training of healthcare professionals must meet the Brazilian Healthcare system’s needs, such as strengthening it and ensuring its full functioning in terms of prevention, promotion, treatment and rehabilitation practices. Healthcare needs to integrate technical and scientific knowledge, such as considering the subjective dimension, which influences the health process, considering the ways of living and working of social subjects^(1)^.

Far beyond the idea of a factor, the social here refers to structuring society according to the mode of production and processes of social construction of biological attributes, such as sex, race and age, reinterpreted as gender, ethnicity and generation. This would imply the insertion of subjects and groups into social classes and other strata, such as gender, race-ethnicity and generation, which, when intersectionalized, will ultimately determine quality of life with its vulnerabilities and potentialities and, consequently, the healthcare profile of subjects and social groups. Thus, health and disease are considered constituent elements of the same process that has the social as a basic determinant, without disregarding individuals’ genotypic and phenotypic biological aspects and the interaction between them and other living beings in nature^(2)^.

Seen in this way, violence also constitutes a social phenomenon and needs to be understood as such, beyond the idea of interaction between subjects through behaviors resulting from individual and isolated initiatives between aggressors and victims. In this context, domestic and gender-based violence is understood as a phenomenon that impacts not only women, but the entire family and, ultimately, society, especially due to its consequences, which extend to the broader social structure itself, such as lost workdays, costs for the health and judicial systems, among others^(2)^.

The Maria da Penha Law, Law 11,340 of August 7, 2016, defines domestic violence as any act or omission based on gender that results in death, injury, physical, sexual, psychological distress, moral or patrimonial damage. This form of violence is recognized as a public health and human rights problem. In this article, we used the concept of gender as socially constructed power relations based on the differences perceived between subjects based on sex, understood as social sex^(2)^.

It is estimated that one in three (35%) women in the world has suffered physical and/or sexual violence perpetrated by a person with whom they have an affective-sexual relationship, generally their partner^(3)^. In Brazil, a survey conducted in 2022 by the *Datafolha* Institute and the Brazilian Public Security Forum, covering 126 municipalities and interviewing people over the age of 16, revealed that approximately 50,000 women suffered some type of violence of this type that year. The highest incidence occurred among black women, with a prevalence of 48%, compared to 33% of the general population. Among the women who reported having suffered violence, 45% did not seek help; 38% believed they could deal with the problem alone; and 21.3% did not report it due to a lack of trust in the police^(4)^. Concerning the prevalence of this problem in Brazil, research carried out with 34,334 women aged between 40 and 59 years showed that 7.6% of them reported having suffered some type of intimate partner violence, 7.07% of which was psychological, 2.75% physical, and 0.68% sexual^(5)^.

The Maria da Penha Law has been an important milestone in tackling domestic and gender-based violence. The health sector is part of a network for providing assistance and addressing this violence, mainly because women often seek healthcare services for problems arising from this phenomenon, such as trauma, sexually transmitted infections, unwanted pregnancies and mental health problems.

In order for the issues of gender inequalities and domestic and gender-based violence to be included in healthcare, they must be addressed in the training of healthcare professionals and, on a recurring basis, in services, through continuing education. We started from the understanding that the issue of domestic and gender-based violence in the health field should not be understood as a specific diagnosis for intervention, but as a context of praxis to recognize social contradictions and guide human action through shared decisions, called “good practices”^(5)^.

In 2022, the first author of this article received an invitation from the municipal interlocutor for Comprehensive Healthcare for People in Situations of Violence in the city of São Paulo to begin supervision as a continuing education strategy for healthcare professionals working in Violence Prevention Centers (VPCs).

Supervision aimed to discuss, in a reflective manner, the practice of providing assistance to people in situations of violence and to build strategies to identify potentialities, difficulties and limitations of assistance, in order to promote space for epistemological curiosity aiming at reinvention of care^(5)^.

## OBJECTIVES

To report the implementation of supervision in violence in two regions of the city of São Paulo in 2022.

## METHODS

In 2015, the healthcare services of the city of São Paulo implemented a line of care for people in situations of violence as a strategy to consolidate the Municipal Policy for Comprehensive Healthcare for People in Situations of Violence. Based on this line of care, VPCs were established in healthcare services, structured with the participation of healthcare professionals from the services themselves, including management. VPCs are expected to work together with professionals to plan and assist people and families in situations of violence in the territory in a cooperative, interprofessional and intersectoral manner.

Supervision took place from March to December 2022, in two regions in southern São Paulo, here named A and B, totaling ten meetings in each region. The meetings were held in person at the headquarters of health coordinators, lasting one hour and 30 minutes each, with an average participation of 50 professionals in each meeting.

In region A, professionals from 30 Basic Health Units (BHUs), four Psychosocial Support Centers (In Portuguese, *Centros de Apoio Psicossocial* - CAPS), two hospitals, five Outpatient Medical Assistance Centers (In Portuguese, *Assistências Médicas Ambulatoriais* - AMAs) and one Specialized Rehabilitation Center (In Portuguese, *Centro Especializado de Reabilitação* - CER) participated. In region B, professionals from 26 BHUs, three CERs, eight AMAs, three CAPS, one Emergency Care Center, two hospitals, one Long-Term Care Institution, one Specialized STD/AIDS Assistance Service, a team specialized in assisting children and adolescents who are victims and/or witnesses of violence and one Victim Support and Reference Center participated.

As part of supervision planning and execution, in each meeting, a summary of content was produced and shared with participating professionals via a Google Drive file, called “memories”, with access for reading. Scientific texts and support materials were also made available to participants.

The construction of this supervision was mirrored in the “conversation technique” care steps, described by d’Oliveira *et al*.^(6)^, which proposes: welcoming without judgment and avoiding the medicalization of the problem; focusing on the support that healthcare services can offer to women and building care in order to guarantee autonomy; ensuring listening and qualified guidance on rights and on the assistance network services and coping with the problem; giving visibility to the problem in healthcare services; knowing how to ask and identify the problem without establishing an interrogation; not disqualifying complaints; and being careful not to reproduce institutional violence.

To work on the content of this conversation technique, a critical-emancipatory perspective was adopted, in order to promote understanding of the reality of the violence suffered, its relationship with the health needs of women and others involved, and the shared construction of paths for possible changes^(7)^.

The meetings were based on dialogue to promote the recognition and problematization of violence as a result of social phenomena, exposing everyday contradictions, dispelling ideological opinions and practices, encouraging reflection on concrete reality in favor of changes and the adoption of new assistance practices^(7)^.

Based on planning, the dynamics and topics were modified according to collective needs, adopting the dialectical method of exposition and analysis. The purpose was to ensure multiple approaches to the object throughout the process.

There were ten meetings in each region. After each meeting, the subsequent meeting involved discussing a case that included the topics previously discussed. To ensure that the topics appeared in the discussions, a script was drawn up to organize the important information and guide the construction of the case to be discussed, taking into account users’ needs and demands with (or without) their support networks, taking care not to judge, naturalize or trivialize the violence suffered by users, in addition to ensuring privacy and confidentiality^(6)^.

Since this experience report did not involve people, it did not require submission to a Research Ethics Committee (REC) and, consequently, the signing of Informed Consent Form. However, it is important to emphasize that all values and standards of ethics in research and intervention were strictly respected.

## RESULTS

Below are the main topics covered and how they were systematized during the supervision process:

### Approaching the object: domestic and gender violence

This first moment aimed to integrate the people in the group so that they could know each other and establish cooperative relationships to exchange knowledge and experiences. The purpose of the meetings and how they would take place were explained. Afterwards, a dynamic was carried out using the game “*No lugar dela*”, which tells stories of women in situations of violence and invites participants to make decisions to address the problem. The following topics were highlighted through the game: a) the importance of detecting violence and understanding how it impacts women’s health and lives; b) the importance of listening to them to understand the paths already taken to address the problem; c) identifying the services already sought and the help obtained or not; d) listening without judging and how not to turn listening into automatic referrals; e) ensuring privacy and confidentiality of cases; f) building a bond between women and the service.

### Conceptual alignment: gender, violence and human rights

Another highlight was aligning the understanding of the concepts of domestic and gender violence, human rights and gender relations and their articulation with healthcare. To this end, articles were made available for prior reading to articulate knowledge with daily experiences in care in the territory and in relation to women assisted. Although one of the meetings exclusively addressed the topic of “gender, violence and human rights”, in all the others, this knowledge was retrieved to promote reflection and, thus, allow successive approaches to the general topic of supervision.

### How to ask about violence experienced

This meeting addressed the impact of violence on women’s health, relating it to the most common complaints that appear in healthcare services. Based on the booklet^(8)^ “*O que devem saber os profissionais de saúde para promover os direitos e a saúde das mulheres em situação de violência*”, the importance of routinely questioning women to detect warning signs of the problem was highlighted. It was discussed how questions can be asked to detect situations or signs of violence, taking into account that the topic should be approached in a sensitive and delicate manner both in groups and individually. Strategies were exercised to ask questions, directly or indirectly, about the violence suffered by users and how the dialogue about the difficulties and facilities of this content can compose care practice. This activity aimed to encourage the search for information about violence in professionals’ healthcare practice, mainly through the identification of frequent signs and symptoms in the statements of women who experience these situations.

### How to develop a care plan

In this meeting, a situation was presented involving one of the characters in the game “*No lugar dela*” so that participants, in groups, could build an assistance plan considering the desires, experiences and possibilities to face the problem of violence. For this, the instrument Singular Therapeutic Project, a device of the Brazilian National Humanization Policy, was used as a facilitator, which proposes to build and share with the people assisted possible assistance paths. The objective was to relate women’s health needs in the face of the violence suffered by a person in the case, with a focus on dialogue about the purpose of health work. The purpose was to reflect on intersectoral care, fostering dialogue between representatives of the services participating in supervision. Subsequently, there was reflection on intersectoral work in the territory or in the care network for people in situations of violence, in order to guarantee access to services and avoid revictimization of users in the process of seeking help. The proposal for direct dialogue with network services contributes to the construction of a common care project, strengthening network work and enhancing support for women in situations of violence^(9)^.

### Attention to safety plan

This meeting aimed to guide the analysis of life-threatening situations experienced by women and to train professionals to guide and support them in developing and implementing a safety plan. Based on the booklet^(8)^ “*O que devem saber os profissionais de saúde para promover os direitos e a saúde das mulheres em situação de violência*”, professionals worked in groups to discuss the topic and the guidance to be provided to women. There was an exchange of knowledge about services that make up the network and their assistance purposes, as prescribed in the Maria da Penha Law, including public safety, justice, social assistance and health.

### Continuing education and health surveillance

The last meeting addressed the consolidation of continuing health education actions and how the information collected at the meetings could serve as input for improving healthcare. The role of healthcare professionals in early diagnosis and reporting of situations of violence was discussed as well as in sharing information with other professionals and services.

### Assessment

Throughout the process, partial assessments were made regarding the topics discussed, asking participants to express their opinions on the matter. Moreover, in the ninth meeting, the game “*No lugar dela*” was used again to detect changes already perceived in the way of thinking about violence, as well as the possibility of new approaches to addressing the problem in healthcare services, in partnership with the intersectoral network. The final assessment allowed professionals to explain their perceptions of the experience, which was unanimously considered very valid and capable of transforming the daily practice of healthcare services.

## DISCUSSION

The meetings focused on critically reflecting on domestic and gender violence as an important barrier to continuing a life without violence and that coping must be guided by a cohesive and integrated practice that guarantees women access to information, the search for exercising their rights and support to face the problem.

The use of playful strategies, such as games, facilitates understanding of acts that characterize domestic and gender-based violence, and discusses the difficulties women experience in their relationships with formal networks (justice, health, social assistance, public safety) or informal networks (friends and family) for coping with violence. The importance of these networks in paths taken by women was highlighted, as support for services, including healthcare services, to provide guidance on institutions to seek and women’s rights.

Due to the diversity of situations that need to be faced when dealing with violence, the games used helped to broaden professionals’ perception of the phenomenon, in order to highlight the fact that reporting the issue alone does not solve the problem and that coping with violence requires a strong support network that can be activated by women. Another point of dialogue reinforced the impossibility of the problem being dealt with in a bureaucratic manner, with a quick solution^(10)^.

### Study limitations

One of the limitations of this study is that it resulted from a practical professional experience, without direct association with previously structured research, with the approval of REC for data collection during supervision. This absence may limit the understanding of the experiences and perspectives of those involved in the process, which would be enriching for a more in-depth analysis.

### Contributions to health, nursing, or public policy

Establish supervision of domestic and gender-based violence, integrating this issue as part of the responsibility of healthcare professionals, especially nursing professionals, to build joint and shared care actions in tackling it.

## FINAL CONSIDERATIONSS

Supervision was planned to reflect and discuss domestic and gender-based violence suffered by women and how to deal with this demand in healthcare services. The purpose of this type of supervision is to provide paths for reflection, innovation and possible incorporation of the gender perspective in the care of people and to build prevention strategies not only at the individual level, but also at the collective level.

The meetings allowed for the equalization of concepts, discussion of cases, sharing of experiences and development of strategies to cope with violence in healthcare practice in health units. The professionals were able to reframe the barriers to give visibility to domestic and gender-based violence, understanding the importance of recognizing the woman involved as the leading actor in care planning.

The collective construction of knowledge and exchange of experiences were fundamental to strengthening and highlighting paths for networking to ensure qualified and humanized assistance to women in situations of violence. The continuity of continuing education actions and health surveillance is essential to keep professionals updated and motivated to cope with domestic and gender-based violence.

Furthermore, it is worth noting that domestic and gender-based violence are highly prevalent in our society, which means that the problem must also be common among women working in healthcare. Empowering them to look at the problem and build possible ways to address it can also represent an important path towards personal change, with the appreciation of experiences and recovery of the notion that this problem is not individual, but rather affects society as a whole and as such must be addressed.

## Data Availability

Not applicable.
