# Supplementary material for: Continuing education to combat domestic and gender-based violence: an experience report
Source: Rev Bras Enferm. 2025 Aug 8;78(Suppl 2):e20240338. doi: 10.1590/0034-7167-2024-0338 (PMC12334134; doi:10.1590/0034-7167-2024-0338)
Supplement: Translation

# Translation: Educação permanente para o enfrentamento da violência doméstica e de gênero: relato de experiência

**DOI:** 10.1590/0034-7167-2024-0338pt

**Authors:** Maria Fernanda Terra, Rosa Maria Godoy Serpa da Fonseca

**Affiliations:** I Universidade de São Paulo. São Paulo, São Paulo, Brasil

**Keywords:** Supervisão de Enfermagem, Enfermagem, Equidade de Gênero, Violência Contra a Mulher, Educação Permanente

## Abstract

**Objetivos:**

relatar a implementação de supervisão para profissionais de saúde dos Núcleos de Prevenção da Violência em serviços de saúde em duas regiões da cidade de São Paulo, Brasil.

**Métodos:**

relato de experiência do planejamento e execução dos encontros de supervisão na perspectiva da educação crítico-emancipatória.

**Resultados:**

foram realizados 20 encontros com reflexões críticas sobre a violência doméstica e de gênero como barreira à continuidade da vida e a necessidade de visibilização e consideração do fenômeno no planejamento da assistência à saúde.

**Considerações Finais:**

a experiência oferece uma perspectiva prática da supervisão aos trabalhadores de saúde coerente com a manutenção da educação permanente e da vigilância em saúde para aprimorar o enfrentamento da violência doméstica e de gênero nos serviços de saúde.

## INTRODUÇÃO

A formação de profissionais de saúde deve atender às necessidades do Sistema Único de Saúde, como fortalecê-lo e garantir o seu pleno funcionamento no que tange às práticas de prevenção, promoção, tratamento e reabilitação. A assistência à saúde precisa integrar conhecimentos técnicos e científicos, como contemplar a dimensão subjetiva, que influencia o processo de saúde, considerando os modos de viver e trabalhar dos sujeitos sociais^(1)^.

Muito além da ideia de fator, o social aqui se refere à estruturação da sociedade de acordo com o modo de produção e os processos de construção social de atributos biológicos, como sexo, raça e idade, ressignificados como gênero, etnicidade e geração. Isso implicaria a inserção dos sujeitos e grupos em classes sociais e em outros estratos, como gênero, raça-etnia e geração, o que, interseccionalizado, em última instância, vai determinar a qualidade de vida com suas vulnerabilidades e potencialidades e, consequentemente, o perfil de saúde dos sujeitos e grupos sociais. Assim, a saúde e a doença são consideradas elementos constituintes de um mesmo processo que tem o social como determinante básico, sem desconsiderar os aspectos biológicos genotípicos e fenotípicos dos indivíduos e a interação entre eles e outros seres vivos da natureza^(2)^.

Visto dessa maneira, a violência também constitui um fenômeno social e precisa ser entendido como tal, para além da ideia da interação entre sujeitos por meio de comportamentos resultantes de iniciativas individuais e isoladas entre agressores e agredidos. Nessa esteira, compreende-se a violência doméstica e de gênero como fenômeno que impacta não apenas as mulheres, mas toda a família e, em última instância, a sociedade, especialmente pelas suas consequências, que se estendem até a própria estrutura social mais ampla, como a perda de dias de trabalho, custos para os sistemas de saúde, judiciário, entre outros^(2)^.

A Lei Maria da Penha, Lei n^o^ 11.340, de 7 de agosto de 2016, define violência doméstica como qualquer ato ou omissão baseado no gênero que resulte em morte, lesão, sofrimento físico, sexual, psicológico, dano moral ou patrimonial. Essa forma de violência é reconhecida como um problema de saúde pública e de direitos humanos. Neste artigo, utilizamos o conceito de gênero como relações de poder socialmente construídas a partir das diferenças percebidas entre os sujeitos com base no sexo, entendido como sexo social^(2)^.

Estima-se que uma em cada três (35%) mulheres no mundo já sofreu violência física e/ou sexual perpetrada pela pessoa com quem estabelece relações afetivo-sexuais, em geral, o companheiro^(3)^. No Brasil, pesquisa realizada em 2022 pelo Instituto Datafolha e Fórum Brasileiro de Segurança Pública, abrangendo 126 municípios e entrevistando pessoas com idade superior a 16 anos, revelou que aproximadamente 50 mil mulheres sofreram alguma violência deste tipo naquele ano. A maior incidência ocorreu entre mulheres negras, com prevalência de 48%, em comparação com os 33% da população em geral. Entre as mulheres que relataram ter sofrido violência, 45% não procuraram ajuda; 38% acreditavam que poderiam lidar sozinhas com o problema; e 21,3% não denunciaram por falta de confiança na polícia^(4)^. Quanto à prevalência do problema no Brasil, pesquisa realizada com 34.334 mulheres com idade entre 40-59 anos mostrou que 7,6% delas referiram ter sofrido algum tipo de violência por parceiro íntimo, sendo 7,07% psicológica, 2,75%, física, e 0,68%, sexual^(5)^.

A Lei Maria da Penha tem sido um marco importante no enfrentamento da violência doméstica e de gênero. O setor da saúde compõe a rede de assistência e de enfrentamento dessas violências, principalmente pelo fato de as mulheres buscarem intensamente os serviços de saúde com problemas decorrentes desse fenômeno, como traumas, infecções sexualmente transmissíveis, gravidez indesejada e problemas de saúde mental.

Para que os temas das desigualdades de gênero e da violência doméstica e de gênero componham a assistência à saúde, faz-se necessário que sejam abordados na formação dos profissionais de saúde e, de modo recorrente, nos serviços, por meio da educação permanente. Partimos da compreensão de que o tema da violência doméstica e de gênero no campo da saúde não deve ser compreendido como diagnóstico específico para a intervenção, mas como contexto de práxis para reconhecer as contradições sociais e orientar a ação humana através de decisões compartilhadas, nominadas “boas práticas”^(5)^.

No ano de 2022, a primeira autora deste artigo recebeu convite da interlocução municipal da Atenção Integral à Saúde da Pessoa em Situação de Violência do município de São Paulo para iniciar a supervisão enquanto estratégia de educação permanente junto a profissionais de saúde que atuavam nos Núcleos de Prevenção à Violência (NPVs).

A supervisão teve como objetivo dialogar, de modo reflexivo, sobre a prática assistencial às pessoas em situação de violência e construir estratégias para identificar potencialidades, dificuldades e limitações da assistência, de modo a promover espaço para a curiosidade epistemológica visando à reinvenção do cuidado^(5)^.

## OBJETIVOS

Relatar a implantação da supervisão em violência em duas regiões do município de São Paulo no ano de 2022.

## MÉTODOS

Nos serviços de saúde do município de São Paulo, em 2015, foi implantada a linha de cuidado para a atenção às pessoas em situação de violência como estratégia para consolidar a Política Municipal de Atenção Integral à Saúde das Pessoas em Situação de Violência. A partir dessa linha de cuidado, foram instituídos os NPVs nos serviços de saúde, estruturados com a participação de profissionais de saúde dos próprios serviços, incluindo a gerência. É esperado que os NPVs atuem em conjunto com os profissionais para planejar e assistir pessoas e famílias em situação de violência no território, de modo cooperativo, interprofissional e intersetorial.

A supervisão aconteceu no período de março a dezembro de 2022, em duas regiões da zona sul da cidade de São Paulo, aqui nominadas por A e B, totalizando dez encontros em cada região. Os encontros foram realizados nas sedes das coordenadorias de saúde presencialmente, com duração de uma hora e 30 minutos cada, com a participação média de 50 profissionais em cada encontro.

Na região A, participaram profissionais de 30 Unidades Básicas de Saúde (UBS), quatro Centros de Apoio Psicossocial (CAPS), dois hospitais, cinco Assistências Médicas Ambulatoriais (AMAs) e um Centro Especializado de Reabilitação (CER). Na região B, participaram profissionais de 26 UBS, três CERs, oito AMAs, três CAPS, um Pronto Atendimento, dois hospitais, uma Instituição de Longa Permanência, um Serviço de Assistência Especializada DST/Aids, uma equipe especializada no atendimento a crianças e adolescentes vítimas e/ou testemunhas de violência e um Centro de Referência e Apoio à Vítima.

Como parte do planejamento e execução da supervisão, em cada encontro, foi produzida uma síntese dos conteúdos que foram compartilhados com os profissionais participantes via arquivo do Google Drive, denominado “memórias”, com acesso para leitura. Foram disponibilizados também textos científicos e materiais de apoio para os participantes.

A construção desta supervisão se espelhou nos passos assistenciais da “técnica de conversa”, descrita por d’Oliveira *et al*.^(6)^, que propõe: acolher sem julgamento e evitar a medicalização do problema; focar nos apoios que os serviços de saúde podem oferecer para a mulher e construir o cuidado, de modo a garantir a autonomia; garantir escuta e orientação qualificada sobre direitos e sobre os serviços da rede assistencial e de enfrentamento para o problema; dar visibilidade ao problema nos serviços de saúde; saber perguntar e identificar o problema sem que se estabeleça um interrogatório; não desqualificar as queixas; e atentar para não reproduzir a violência institucional.

Para trabalhar o conteúdo dessa técnica de conversa, foi adotada a perspectiva crítica-emancipatória, de modo a promover a compreensão da realidade da violência sofrida, sua relação com as necessidades de saúde das mulheres e demais envolvidos, e a construção compartilhada de caminhos para as mudanças possíveis^(7)^.

Os encontros se baseavam no diálogo para promover o reconhecimento e a problematização da violência como resultante dos fenômenos sociais, expondo contradições cotidianas, desfazendo opiniões e práticas ideológicas, incentivando a reflexão sobre a realidade concreta em prol de mudanças e adoção de novas práticas assistenciais^(7)^.

Tendo como base o planejamento, a dinâmica e os temas se modificavam conforme a necessidade coletiva, adotando-se o método dialético de exposição e análise. A finalidade era garantir múltiplas abordagens do objeto ao longo do processo.

Foram dez encontros em cada região. Após cada um, o encontro subsequente envolvia discutir um caso agregando os temas tratados anteriormente. Para garantir que os temas aparecessem nas discussões, foi elaborado um roteiro para organizar as informações importantes e orientar a construção do caso a ser discutido, levando em conta as necessidades e demandas das usuárias com (ou não) suas redes de apoio, com o cuidado de não julgar, naturalizar ou banalizar a violência sofrida pelas usuárias, além de garantir a privacidade e o sigilo^(6)^.

Por não envolver pessoas, este relato de experiência dispensou a submissão ao Comitê de Ética em Pesquisa (CEP) e, consequentemente, a assinatura do Termo de Consentimento Livre e Esclarecido. No entanto, cabe ressaltar que foram rigorosamente respeitados todos os valores e normas de ética em pesquisa e intervenção.

## RESULTADOS

A seguir, estão os principais temas abordados e como foram sistematizados durante o processo de supervisão:

### Aproximação do objeto: a violência doméstica e de gênero

Este primeiro momento teve como objetivo integrar as pessoas do grupo para que se conhecessem e estabelecessem relações de cooperação para troca de conhecimentos e experiências. Foi explicada a finalidade dos encontros e como aconteceriam. Posteriormente, foi feita uma dinâmica usando o jogo “No lugar dela”, que narra histórias de mulheres em situação de violência e convida os participantes para tomarem decisões para enfrentar o problema. A partir do jogo, foram destacados os seguintes temas: a) importância da detecção da violência e a compreensão de como impacta a saúde e a vida das mulheres; b) a importância de ouvi-las para entender caminhos já traçados para enfrentar o problema; c) identificar os serviços já buscados e a ajuda conseguida ou não; d) ouvir sem julgar e como não transformar a escuta em encaminhamento automático; e) garantir privacidade e sigilo dos casos; f) construir vínculo entre as mulheres e o serviço.

### Alinhamento conceitual: gênero, violência e direitos humanos

Outro ponto de destaque foi alinhar o entendimento dos conceitos de violência doméstica e de gênero, direitos humanos e relações de gênero e sua articulação com a assistência à saúde. Para tanto, foram disponibilizados artigos para leitura prévia para articular os conhecimentos às experiências cotidianas na assistência no território e em relação às mulheres assistidas. Apesar de um dos encontros ter exclusivamente tratado do tema “gênero, violência e direitos humanos”, em todos os demais, esses conhecimentos eram resgatados para promover a reflexão e, assim, permitir aproximações sucessivas ao tema geral da supervisão.

### Como perguntar sobre a violência sofrida

Neste encontro, foi tratado o tema dos impactos da violência sobre a saúde das mulheres, relacionando-os às queixas mais comuns que aparecem nos serviços de saúde. Com base na cartilha^(8)^ “O que devem saber os profissionais de saúde para promover os direitos e a saúde das mulheres em situação de violência”, foi destacada a importância de questionar, de forma rotineira, as mulheres para detectar os sinais de alerta para o problema. Foi discutido como as perguntas podem ser feitas para detectar situações ou indícios de violência, levando em conta que o tema deve ser abordado de forma sensível e delicada tanto nos grupos quanto individualmente. Foram exercitadas estratégias para fazer as perguntas, de maneira direta ou indireta, sobre a violência sofrida pelas usuárias e como o diálogo sobre as dificuldades e facilidades desse conteúdo pode compor a prática assistencial. Essa atividade teve como finalidade propiciar a busca de informações sobre a violência na prática assistencial dos profissionais, principalmente mediante a identificação de sinais e sintomas frequentes nas falas das mulheres que vivenciam essas situações.

### Como elaborar o plano de cuidado

Neste encontro, foi apresentada uma situação de uma das personagens do jogo “No lugar dela”, para que os participantes, em grupos, construíssem um plano assistencial considerando os desejos, vivências e possibilidades para enfrentar o problema da violência. Para isso, foi utilizado o instrumento Projeto Terapêutico Singular, dispositivo da Política Nacional de Humanização, como facilitador, que propõe construir e compartilhar com as pessoas assistidas caminhos assistenciais possíveis. O objetivo foi relacionar as necessidades de saúde das mulheres diante da violência sofrida pela personagem do caso, com foco no diálogo sobre a finalidade do trabalho em saúde. A finalidade foi refletir sobre a assistência de forma intersetorial, propiciando diálogo entre os representantes dos serviços participantes da supervisão. Posteriormente, houve reflexão sobre o trabalho intersetorial no território ou na rede assistencial às pessoas em situação de violência, de modo a garantir o acesso aos serviços e evitar a revitimização das usuárias no processo de busca por ajuda. A proposição do diálogo direto com os serviços da rede contribui para que se construa um projeto assistencial comum, fortalecendo o trabalho em rede e potencializando o suporte às mulheres em situação de violência^(9)^.

### Atenção ao plano de segurança

Este encontro teve como objetivo orientar a análise das situações de risco de morte vivenciadas pelas mulheres e capacitar os profissionais para orientá-las e apoiá-las na elaboração e concretização de um plano de segurança. Com base na cartilha^(8)^ “O que devem saber os profissionais de saúde para promover os direitos e a saúde das mulheres em situação de violência”, os profissionais trabalharam em grupos para discutir o tema e as orientações a serem fornecidas às mulheres. Houve troca de conhecimentos sobre os serviços componentes da rede e suas finalidades assistenciais, conforme prescrito na Lei Maria da Penha, incluindo segurança pública, justiça, assistência social e saúde.

### Educação permanente e vigilância em saúde

O último encontro tratou da consolidação de ações de educação permanente em saúde e de como as informações coletadas nos encontros poderiam servir de insumo para melhorar a atenção à saúde. Foi discutido o papel dos profissionais de saúde no diagnóstico precoce e na notificação das situações de violência, bem como no compartilhamento das informações com outros profissionais e serviços.

### Avaliação

Durante todo o processo, foram feitas avaliações parciais a respeito dos temas tratados, solicitando que os participantes se posicionassem a respeito. Além disso, no nono encontro, foi aplicado novamente o jogo “No lugar dela” para detectar as mudanças já percebidas no modo de pensar a violência, assim como a possibilidade de novas abordagens para o enfrentamento do problema nos serviços de saúde, em parceria com a rede intersetorial. A avaliação final permitiu que os profissionais explanassem suas percepções sobre a experiência, que foi unanimemente considerada muito válida e passível de transformar a prática cotidiana dos serviços de saúde.

## DISCUSSÃO

O foco dos encontros foi refletir criticamente sobre a violência doméstica e de gênero como uma barreira importante para a continuidade da vida sem violência e que o enfrentamento deve ser pautado em uma prática coesa e integrada que garanta o acesso das mulheres à informação, à busca pelo exercício dos seus direitos e ao suporte para enfrentar o problema.

O uso de estratégias lúdicas, como os jogos, propicia entender os atos que caracterizam a violência doméstica e de gênero, e dialogar sobre as dificuldades vividas pelas mulheres nas suas relações com as redes formais (justiça, saúde, assistência social, segurança pública) ou informais (amigos e familiares) de enfrentamento da violência. Ressaltava-se a importância dessas redes nos caminhos percorridos pelas mulheres, como suporte dos serviços, inclusive os de saúde, para orientar sobre instituições a serem buscadas e os direitos das mulheres.

Pela diversidade de situações a serem enfrentadas para lidar com a violência, os jogos utilizados contribuíram para ampliar a percepção dos profissionais sobre o fenômeno, de modo a trazer para a cena o fato de que a denúncia por si só não resolve o problema e que o enfrentamento da violência exige uma rede de apoio forte a ser acionada pelas mulheres. Outro ponto de diálogo reforçou a impossibilidade de que o problema seja tratado de maneira burocrática, com rápida solução^(10)^.

### Limites do estudo

Um dos limites deste estudo é que ele resultou de uma experiência profissional prática, sem associação direta com uma pesquisa previamente estruturada, com a aprovação de CEP para a coleta de dados durante a supervisão. Essa ausência pode limitar a compreensão das experiências e perspectivas dos envolvidos no processo, o que seria enriquecedor para uma análise mais profunda.

### Contribuições para as áreas da saúde, enfermagem, ou políticas públicas

Instituir a supervisão em violência doméstica e de gênero, integrando essa questão como parte da responsabilidade dos profissionais de saúde, especialmente profissionais de enfermagem, para construir ações assistenciais conjuntas e compartilhadas no enfrentamento.

## CONSIDERAÇÕES FINAIS

A supervisão foi planejada para refletir e discutir sobre a violência doméstica e de gênero sofrida pelas mulheres e de como lidar com essa demanda nos serviços de saúde. A finalidade desse tipo de supervisão é propiciar caminhos para a reflexão, inovação e possível incorporação da perspectiva de gênero no cuidado das pessoas e construir estratégias de prevenção não apenas na esfera individual, mas coletiva.

Os encontros permitiram equalizar conceitos, discutir casos, compartilhar experiências e elaborar estratégias para o enfrentamento da violência na prática assistencial das unidades de saúde. Os profissionais puderam ressignificar as barreiras para dar visibilidade à violência doméstica e de gênero, compreendendo a importância de reconhecer a mulher envolvida como protagonista no planejamento do cuidado.

A construção coletiva de conhecimentos e a troca de experiências foram fundamentais para fortalecer e pontuar caminhos para o trabalho em rede para garantir uma assistência qualificada e humanizada às mulheres em situação de violência. A continuidade das ações de educação permanente e a vigilância em saúde são essenciais para manter os profissionais atualizados e motivados para o enfrentamento da violência doméstica e de gênero.

Além disso, vale ressaltar que a violência doméstica e de gênero tem alta prevalência na nossa sociedade, o que significa que o problema também deve ser comum entre as exercentes do trabalho em saúde. Capacitá-las para olhar para o problema e construir caminhos possíveis para o seu enfrentamento pode significar um caminho importante também de mudança pessoal, com valorização de experiências e resgate da noção de que esse problema não é individual, mas de toda a sociedade e como tal deve ser enfrentado.

## Data Availability

Não se aplica.
